# Optimizing Care for Adults with Congenital Heart Disease: Results of a Conjoint Analysis Based on a Nationwide Sample of Patients Included in the German National Register

**DOI:** 10.3390/jcm10163483

**Published:** 2021-08-06

**Authors:** Astrid E. Lammers, Paul C. Helm, Ulrike M. Bauer, Ann-Kathrin van Huelsen, Helmut Schneider, Helmut Baumgartner, Gerhard-Paul Diller

**Affiliations:** 1 Department of Cardiology III—Adult Congenital and Valvular Heart Disease, University Hospital Muenster, Albert-Schweitzer Campus 1, 48149 Münster, Germany; helmut.baumgarter@ukmuenster.de; 2 Department of Paediatric Cardiology, University Hospital Münster, 48149 Münster, Germany; 3 National Register for Congenital Heart Defects, Augustenburger Platz 1, 13353 Berlin, Germany; helm@kompetenznetz-ahf.de (P.C.H.); ubauer@kompetenznetz-ahf.de (U.M.B.); 4 DZHK (German Centre for Cardiovascular Research), Potsdamer Straße 58, 10785 Berlin, Germany; 5 GfK, Uhlentwiete 14, 20355 Hamburg, Germany; a.veenendaal@web.de; 6 Department of Marketing, Steinbeis-Hochschule, Ernst-Augustin-Straße 15, 12489 Berlin, Germany; h.schneider@steinbeis-smi.de

**Keywords:** adult congenital heart disease, healthcare provision, conjoint analysis, registry, survey

## Abstract

(1) Background: Congenital heart disease (CHD) requires lifelong specialized care. Failure to follow up and gaps in care are common in this group and lead to increased morbidity/mortality. We evaluated patients’ perceived needs and expectations regarding specialized care using state-of-the-art statistical and market research techniques based on a nationwide sample of CHD patients. (2) Methods: A random sample of adults with CHD registered in the German National Register for Congenital Heart Defects were invited to answer an adaptive online questionnaire based on the conjoint analysis (CA) technique. CA determines the relative importance of various aspects of health care provision and allows individuals to trade between characteristics, thus recognizing limited resources. (3) Results: 637 patients participated (mean age 33.8 ± 12.6 years; 55.6% female; disease complexity: simple defect 12.6%, moderate complexity 40.3%, complex CHD 40.2%) in the analysis. Patients assigned the highest relative importance to aspects of patient–physician communication, physician qualifications, waiting time, medical care, and medical equipment. Comfort-related aspects such as driving time or hotel aspects of care received much lower scores. We identified four well-defined clusters of patients with differing expectation patterns: (i) time sensitive patients; (ii) excellence seeking patients; (iii) continuity seekers, and (iv) support seeking patients. (4) Conclusions: Adult CHD patients rank effective patient–physician interaction and communication as the most important factors. As we identified significant heterogeneity between CHD patients, centers should cater for individual preferences and integrate individual needs into treatment plans to prevent failure to follow up and ensure patient compliance.

## 1. Introduction

Congenital heart disease (CHD) is the most common congenital defect in humans [1,2] and requires lifelong specialized care [3,4,5]. Nowadays, about 90% of CHD patients reach adulthood [3,6,7,8,9,10]. However, failure to follow up or gaps in care are common and morbidity and mortality are still unacceptably high in this group [3,11,12,13]. The integration of individual patient needs and preferences into treatment plans could help to prevent failure to follow-up and ensure patient compliance [14,15]. To evaluate subjective patient needs and expectations related to specialized care in CHD, we applied state-of-the-art statistical and market research methods to a sample of adult patients with CHD registered in the German National Register for Congenital Heart Defects. These patients were invited to answer an adaptive online questionnaire based on the conjoint analysis (CA) technique. Conjoint analysis has a strong theoretical basis, is widely used in psychology or market research and is recommended for valuing provision of public services [16,17,18,19,20,21]. It allows the relative importance of various aspects of health care provision to be determined and allows individuals to trade between characteristics, thus recognizing limited resources. Using this technology, we aimed to better understand individual expectations as well as to uncover potential heterogeneity between patients.

## 2. Materials and Methods

The National Register for Congenital Heart Defects (NRCHD) conducted an online survey in cooperation with the Center for Adults with Congenital Heart Defects Muenster and the Steinbeis University Berlin. The survey’s primary objective was to collect information on the patients’ needs and expectations regarding specialized care of adults with congenital heart disease (ACHD) in Germany. With 53,503 members (as of June 2018), the NRCHD is Europe’s largest register of CHD. It is representative of the German cohort of patients with CHD [22]. For patient recruitment, the register’s database was searched for patients who were 18 years or older at the time of the survey and for whom a mail/e-mail address was available. The cardiac diagnoses were coded in accordance with classification of the International Pediatric and Congenital Cardiac Code (IPCC code) [23]. Diagnoses were classified into four severity groups (simple, moderate, complex, others) following Warnes et al. [24]. The patient empowerment score is based on nine individual items. These include three items related to ability to search for information, three items focusing on building up knowledge and three items related to the patient’s subjective active participation in the treatment. An average score was determined for all three sub-aspects and the average patient empowerment score was calculated from these three averages. The psychological/psychiatric comorbidity score is based on four individual items. Two of these four items concern depression and two items focus on the area of anxiety of the interviewed patients. An average score was determined for both sub-aspects and the average psychological/psychiatric comorbidity score was calculated from these two area averages.

### 2.1. Study Design and Questionnaire

First, a sample of adult CHD patients registered in the NRCHD was invited to answer general questions regarding their life and treatment situation. Next, we invited these patients to answer a second questionnaire, consisting of an adaptive online questionnaire based on the CA technique. The CA technique is well-established in marketing research and allows participants to rate the subjective relative importance of various aspects of health care provision. The adaptive questionnaire consists of three steps:

- Step one (Description of the perfect world scenario): The participants were asked to provide information about the perfect scenario regarding an adult CHD doctor’s consultation if no restrictions were present.
- Step two (Testing acceptable trade-offs): Based on the answers given in step one, the participants were asked to limit the selection and to evaluate different scenarios (trade-offs) regarding a doctor’s consultation and to decide whether different scenarios would be acceptable.
- Step three (Combining Steps 1 and 2): Based on the information from steps one and two, participants were presented with three scenarios to choose from. Study participants were asked to choose the most preferable of the remaining options. For in-depth information on the technique employed and the calculations performed, we refer to the published literature [25].

**Figure 1:**
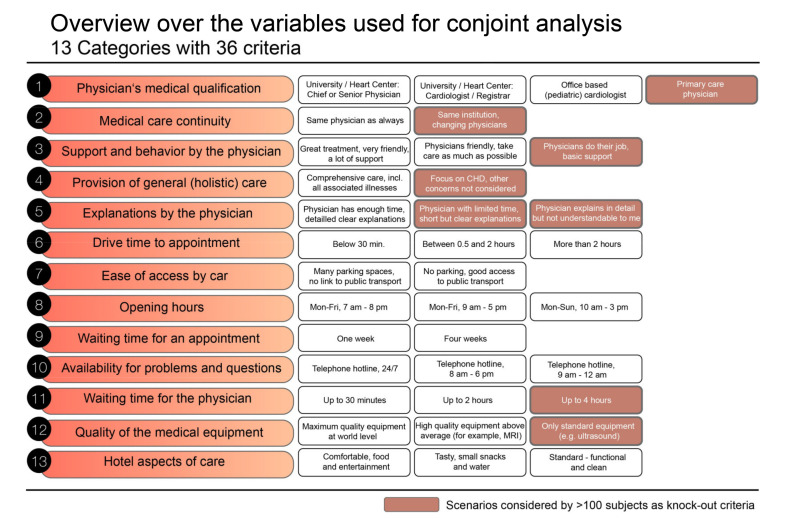
Overview over the 13 categories and their respective subvariables used for conjoint analysis. The rectangles marked in orange were identified as knock-out criteria in the first step of the conjoint analysis (see text for details).

Overall, the participants were asked to estimate the importance of 13 items (with a total of 36 characteristic values) for service provision. Scores are formed to assign a global importance to each of these 13 factors. Parameters included in the CA covered aspects of physicians’ qualification, medical care continuity, support and behavior by physicians, provision of general (holistic) care, explanations provided by the physician, drive time to medical appointment, ease of access by car, availability of services and opening hours, waiting time for an appointment, availability for problems and questions (such as telephone hotline), waiting time to see the physician when in clinic, quality of the available medical equipment and hotel or comfort aspects of care. Figure 1 provides an overview over these parameters and the options available to choose from.

### 2.2. Statistical Analysis

In addition to conventional descriptive statistical analysis (e. g. comparison of means) a cluster analysis was performed, and the conjoint analysis technique (CA) was used. T-tests and analysis of variance (ANOVA) were used for group comparisons for interval scaled data. Alpha error adjustment in multiple testing [26] was not performed due to the study’s conception being explorative and to avoid overlooking potential influencing factors. SPSS (version 22, IBM Computer Systems) was used for statistical analyses.

## 3. Results

**Figure 2:**
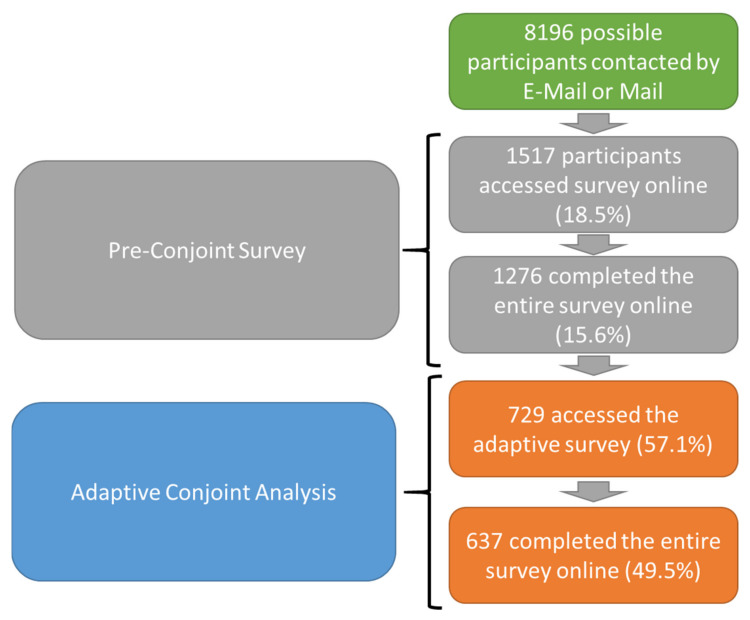
Overview over the study design and the response rates starting from the entire cohort of adult CHD patients with e-mail/mail addresses available to those who participated in the conjoint analysis (see text for details).

Overall, 637 patients were included in the statistical analyses (corrected CA response rate based on pre-screening: 49.5%; mean age 33.8 ± 12.6 years; 55.6% female). For more details regarding the study design and response rates, see Figure 2. Patients were classified based on CHD complexity as having simple defects (12.6%), moderate complexity defects (40.3%), complex CHD (40.2%) and other/unclassified defects (6.9%). Diagnoses covered the entire spectrum of CHD including (amongst others), for example, 41 patients with univentricular hearts, 93 Tetralogy of Fallot patients, 53 patients with a systemic right ventricle, 31 atrioventricular septal defect (AVSD) patients, nine patients with Ebstein anomaly, 60 aortic coarctation, 60 patients with a ventricular septal defect, and 44 patients with an atrial septal defect.

### 3.1. Descriptive Conjoint Analysis

**Figure 3:**
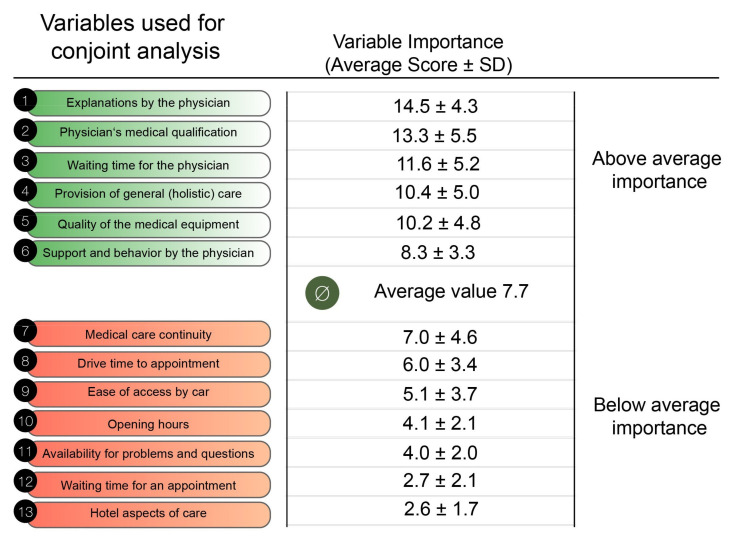
Overview of the importance assigned to each of the 13 categories used for conjoint analysis by the study participants.

Patients assigned the highest relative importance scores to the following aspects of service provision: explanation by the physician (average importance score: 14.5 ± 4.3), physician’s qualifications (average importance score: 13.3 ± 5.5), waiting time for the physician (average importance score: 11.6 ± 5.2), provision of general in addition to CHD focused medical care (average importance score: 10.4 ± 5.9) and quality of the medical equipment (average importance score: 10.2 ± 4.8). In contrast, other aspects such as waiting time for an appointment, driving time or hotel aspects of care received much lower scores (Figure 3).

**Figure 4:**
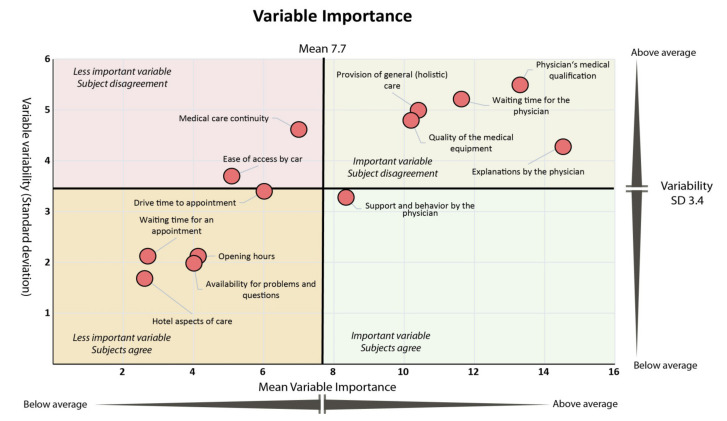
Scatterplot of variables’ average importance (*x*-axis) versus their average variability measured as standard deviation (SD; *y*-axis). The figure shows which variables were not only rated as being of above or below average importance but also how well participants agreed on the importance assigned. For further discussion of the quadrants, see text.

Following the concept that a parameter is more relevant if its average score is higher, but also if there is less disagreement between subjects (i.e., if the variability in the response patterns or standard deviation (SD) of the score is lower) we assessed these two factors in combination. As illustrated in Figure 4, there was consensus between subjects that support, and adequate behavior of physicians is important (right lower quadrant).

While provision of holistic care, quality of medical equipment, physicians’ qualifications, explanations, and lower waiting times during the consultation were also rated as having above average importance, responses showed a higher level of heterogeneity between individuals for these variables (right upper quadrant). The consensus (i.e., lower variability in response patterns) was that hotel aspects of care, opening hours or waiting time for appointment are of limited relevance (left lower quadrant). Medical care continuity or ease of access by car also received lower than average importance scores but response pattern varied more in relation to these parameters (upper left quadrant).

**Figure 5:**
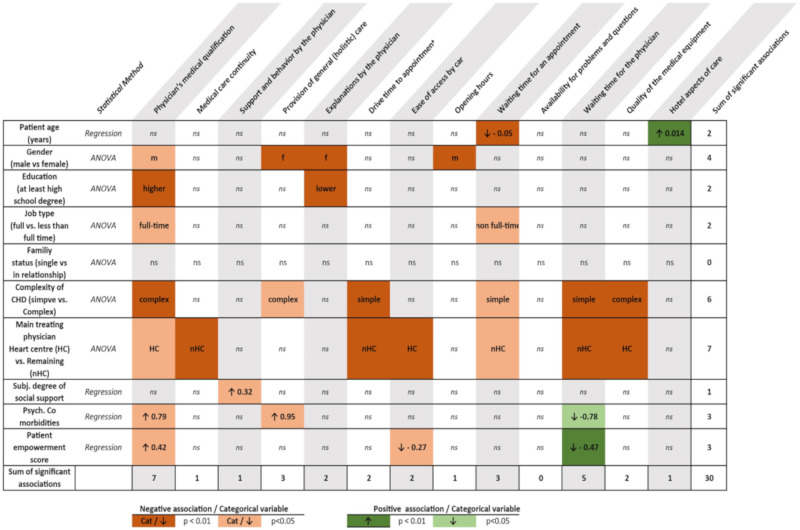
Differences between patients’ preferences and subject characteristics; ns = not significant.

To further investigate potential differences between patients’ preferences and subject characteristics, the impact of age, gender, educational achievement, having a full-time job, CHD complexity as well as available social support, psychiatric co-morbidities and overall patient empowerment on preference profiles were assessed. As shown in Figure 5 and Table A1 (Appendix A), this revealed some interesting associations. For example, females tended to place more emphasis on provision of holistic care and understandable explanations, while males were more interested in the physician’s qualification and more convenient opening hours (*p* < 0.05 for all). Similarly, younger patients paid more attention to shorter waiting times and comfort aspects (*p* < 0.05 for both). Maybe not surprisingly, patients with complex underlying heart defects placed more emphasis on physician’s qualification, a holistic care model and the quality of the available medical equipment, while those with simple underlying defects were more interested in shorter drive time and waiting times for an appointment and for the physician during the consultation (*p* < 0.05 for all). Those patients with increased need for social support and with psychological/psychiatric co-morbidities demanded significantly more physician support and higher medical qualification as well as holistic care, respectively. Patients under follow-up at heart centers also had a different preference profile compared to those seen by office-based colleagues. As illustrated by the row/column sums in Figure 5, the highest degree of heterogeneity is related to physicians’ qualifications (total of seven points = significant associations), followed by waiting time for the physician (five points). The patient characteristics associated with highest response heterogeneity were gender, complexity of CHD and mode of follow-up (four, six and seven points, respectively).

### 3.2. Cluster Analysis

Applying cluster analysis to the data identified the following four well defined clusters of patients with differing expectation patterns:

1. Time-sensitive patients focusing on organizational aspects of care with high preference for quick appointments, short driving and waiting time.
2. Excellence-seeking patients favoring centers with a high level of expertise and 170 state-of-the art technical equipment.
3. Continuity of care cluster of patients seeking to consult the same physician.
4. High level of individual support patients including detailed explanations by health care professionals as well as holistic medical treatment.

**Figure 6:**
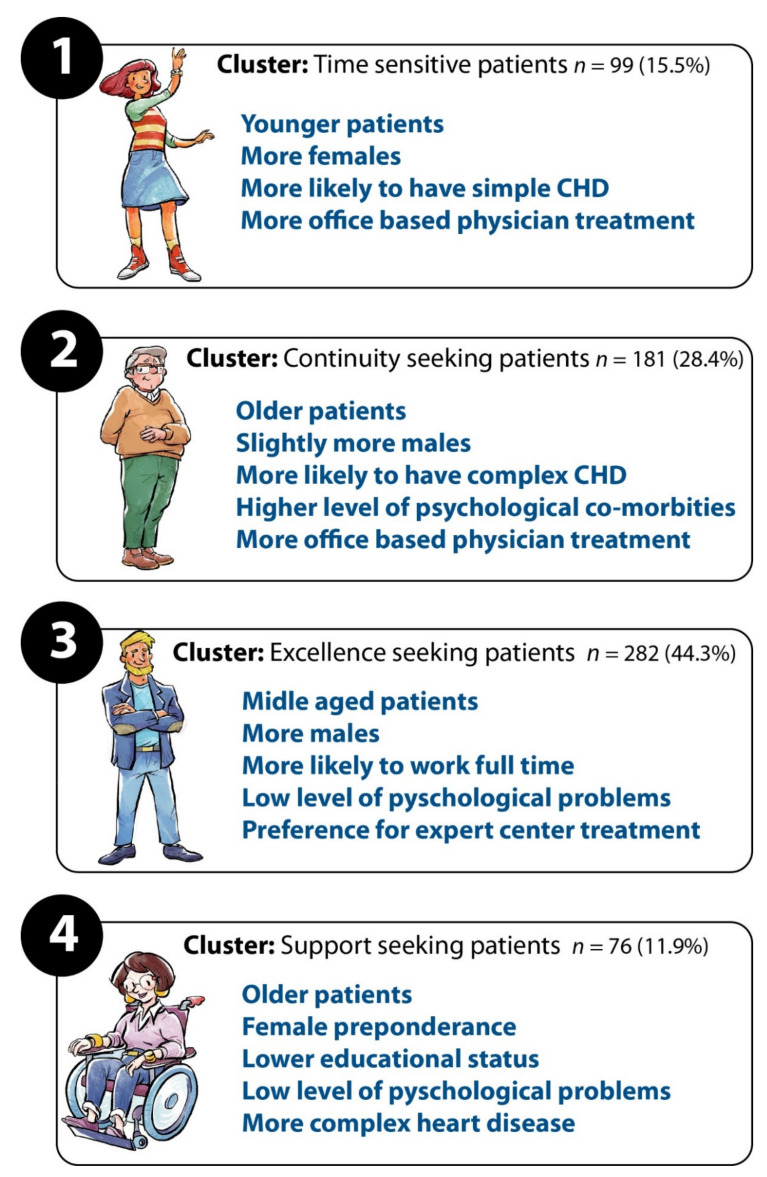
Overview over the four identified clusters.

Results are shown in detail in Table 1. The four groups not only showed a different pattern of personal preferences regarding the 13 studied variables (Table 1) but were also demographically different, as illustrated in Figure 6.

## 4. Discussion

To our knowledge, this is the first study to apply conjoint analysis in the setting of adult CHD. Adult CHD patients are affected by chronic lifelong heart disease and require continuous specialized follow-up [3,9,13]. Lapses in care and failure to follow up are associated with increased complications and mortality [13,27]. Abandoning the paternalistic top down medic–patient paradigm, we aimed to follow a modern marketing approach of considering patients as customers and rational individuals. This approach assumes that patients might be more likely to accept lifelong care if it meets their expectations and needs. Using a large sample of individuals (including >600 patients) drawn from one of the largest global CHD registries [28] and state-of-the art statistical models [20,25], we show that the physician–patient relationship seems to be of greatest significance in this context. Particularly, clear and understandable explanations by the doctor emerged on average as the most important criterion for patients. This is consistent with the more general finding of our study, namely that criteria related to medical treatment clearly dominate those parameters with organizational reference. While accepting that organizational aspects of care are important, this emphasizes the central role of the CHD physician for patients. Patients demand high quality care and expect clear and comprehensive information on their heart condition, a holistic treatment approach, comprehensive physician support and high-quality equipment to be used. In contrast, opening hours, hotel aspects of care or waiting times are far less relevant.

Based on the first stage of the CA method, we found that the main knock-out criteria for patients were primary care physician led treatment only, poor doctor–patient communication (physician has time, but does not explain clearly and understandable), as well as severely prolonged waiting times during consultation (up to 4 h waiting time). In the same context, patients also highlighted what they perceived to be indispensable for adult CHD care. These factors included a comprehensive (holistic) care (including extra-cardiac problems), enough time for consultation and clear and understandable, comprehensive explanations. The main finding of our study, that communication and a good physician–patient relationship are of central importance to patients, is in line with previous studies in the setting of congenital heart disease as shown by publications by Helm et al. and Lesch et al. previously [4,22,29]. Interestingly, patients’ subjective preferences were found to be heterogeneous on further analysis. Not unexpectedly, CHD complexity modulated parameters’ importance. Patients with complex CHD tended to prefer physicians with higher qualifications, more center-based treatment, a holistic treatment approach and demanded high quality medical equipment. In addition, age, gender, educational level, and employment status emerged as significant modulators of preferences. Given this heterogeneity, we investigated whether groups or clusters of patients might exist which exhibit a more homogeneous expectation pattern. This information is helpful for clinicians and hospital administrators to optimize care, attract and maintain patients under follow-up. Using statistical cluster analysis, we identified four distinct groups of patients with differing needs. As illustrated in Figure 6, this included a group of younger patients with less complex CHD who emerged as time-sensitive patients. These patients prefer office-based physicians, probably due to better accessibility and shorter waiting time. These patients are also willing to sacrifice care excellence for convenience. Similarly, another group of continuity seekers seemed to prefer more accessible and potentially more predictable care. This group of patients preferring continuity of care tended to be older, more likely to be male and with a higher proportion of complex CHD alongside a higher level of psychological problems. A third cluster was formed by excellence-seeking individuals. This was the largest of the four clusters. The typical patients in this group are middle-aged males with high educational status, in full-time employment and expecting the optimal quality of care. The fourth group consisted of support-seeking patients, who tended to be older, more likely to be female with more complex CHD and a higher percentage of psychological problems. It should be noted, however, that overlap exists between these clusters and patients may exhibit characteristics of more than one cluster. In addition, the current analysis is cross-sectional and does not account for temporal effects. Thus, it is perceivable that patients may switch groups over time, including as an effect of aging or emerging complications/co-morbidities. Nevertheless, we contend that depicting the four archetypal clusters illustrates the spectrum of patients’ care expectations. By organizing care to cater for all these groups, the needs of most adult CHD patients are likely to be covered. We believe that acknowledging these differences in expectations could be a main element of service planning and treatment provision and may help to increase patient satisfaction and decrease the failure to follow up rate significantly [14,27]. Furthermore, it seems paramount to educate physicians and nurses about the importance of good communication and holistic treatment, as well as fostering communication skills [30]. In particular, treatment continuity (e.g., by offering the option of being seen by the same physician every time in clinic) could be an easily addressable aspect as it ensures that the treating physician understands patients’ needs and creates trust to ensure patient compliance and continuity of medical treatment. Our findings are consistent with previous studies focusing chiefly on the transition period [31] but greatly build on these data by covering a larger age period and by utilizing CA methods. The results reported here could help to improve the clinical practice by delineating the individual patient needs and creating a treatment plan together with the patients to increase compliance and decrease failure to follow up.

### Strengths and Limitations

As stated above, this was a cross-sectional analysis based on a sample of 257 individuals drawn from the National Register for Congenital Heart Defects. Therefore, by design, the study included only patients who were able (availability of electronic communication equipment) and willing to participate. We cannot exclude the possibility that this sample is biased compared to the adult CHD patients in the community. Given the fact that the NRCHD is one of the largest of its kind globally and has been shown to be representative for the CHD spectrum in Germany, as well as the size of the study, we contend that this analysis provides important insights into patients’ preferences and should be considered when planning service provision. Due to the cross-sectional nature of the study, no temporal aspects could be investigated. Demographic information, as well as diagnosis, was cross-matched with the curated underlying registry, ensuring that this information is complete and correct. We did not perform an alpha error correction for significant values in multiple tests, as the focus of the study was mainly descriptive to avoid uncovering any possible group differences. Not all differences between the four clusters depicted in Figure 6 reached a significance level of <0.05, therefore this analysis should be seen as a descriptive illustration of the clusters uncovered. The external validity of the findings and whether and to what extent results can be extrapolated to other international health care systems should be subject to future research. Specifically, as we performed this study in one country, further studies are required to test whether and how the uncovered clusters apply to other geographic areas. Unfortunately, we do not hold data to study which patients were most compliant with scheduled visits or care or which patients clusters were more likely to drop out of care. This should also be the subject of further investigations.

## 5. Conclusions

Among all the aspects of care evaluated, adult CHD patients rank effective patient–physician interaction and communication as the most important factors. In contrast, hotel aspects of care, distance to the CHD center or waiting time are relatively unimportant in general. As we identified significant heterogeneity between CHD patients, heart centers and office-based cardiologists should consider individual patient preferences and integrate individual needs into treatment plans to prevent loss to follow-up and ensure patient compliance.

## Figures and Tables

**Table 1:** Overview of the distribution of the mean ± standard deviation of the 13 characteristics by patient cluster. Appt., appointment; hrs., hours.

Variable	All Subjects	Time	Excellence	Continuity	Support
	*n* = 637	*n* = 100	*n* = 278	*n* = 183	*n* = 76
Physician qualification	13.3 ± 5.5	7.9 ± 3.7	15.7 ± 4.6	14.5 ± 5.2	8.6 ± 3.5
Care continuity	7.0 ± 4.6	4.8 ± 2.9	4.9 ± 2.9	11.9 ± 4.1	5.5 ± 3.4
Support by physician	8.3 ± 3.3	7.0 ± 3.3	8.4 ± 3.2	8.0 ± 3.2	10.5 ± 3.4
Holistic care	10.4 ± 5	9.0 ± 4.7	10.1 ± 4.6	9.1 ± 4.6	16.9 ± 2.7
Explanations	14.5 ± 4.3	15.1 ± 4.4	13.5 ± 3.8	14.6 ± 4.7	17.7 ± 3.6
Drive time	6.0 ± 3.4	9.9 ± 3.5	5.4 ± 2.8	5.7 ± 3.0	4.1 ± 2.2
Car access	5.1 ± 3.7	6.7 ± 4.7	5.0 ± 3.3	4.7 ± 3.2	4.9 ± 4.0
Opening hrs.	4.1 ± 2.1	5.0 ± 2.3	3.9 ± 2.0	4.1 ± 2.2	3.8 ± 2.0
Appt. wait time	2.7 ± 2.1	3.4 ± 2.4	2.6 ± 1.9	2.7 ± 2.3	2.5 ± 1.6
Center availability	4.0 ± 2.0	4.6 ± 2.1	3.7 ± 1.8	4.2 ± 2.1	4.1 ± 1.7
Wait at appt.	11.6 ± 5.2	17.4 ± 3.9	11.3 ± 4.8	10.2 ± 4.5	8.4 ± 4.1
Quality of equipment	10.2 ± 4.8	6.9 ± 3.4	13.1 ± 4.2	7.4 ± 3.6	10.7 ± 4.4
Hotel aspects	2.6 ± 17.0	2.5 ± 1.7	2.5 ± 1.6	2.9 ± 1.9	2.5 ± 1.4

## Data Availability

Data is available on reasonable request via the National Register of Congenital Heart Disease and under the published rules and regulation of the Register.

## Abbreviations

The following abbreviations are used in this manuscript:

CA	Conjoint Analysis
CHD	Congenital heart disease
SD	Standard deviation

## Appendix A

**Table A1:** Statistical comparison between various complexity levels of congenital heart disease, regarding importance of the 13 study parameters. Appt. = appointment; mod. = moderate.

Variable	Simple vs. mod.	Simple vs. Complex	Mod. vs. Complex
Medical qualification	*p* = 0.09	*p* < 0.05	*p* = 0.62
Care continuity	*p* = 0.41	*p* < 0.05	*p* < 0.05
Support by physician	*p* = 0.09	*p* = 0.71	*p* = 0.06
Holistic care	*p* = 0.26	*p* = 0.134	*p* = 0.53
Explanations	*p* = 0.35	*p* = 0.25	*p* = 0.74
Drive time	*p* = 0.08	*p* < 0.01	*p* = 0.26
Car access	*p* = 0.22	*p* = 0.44	*p* < 0.01
Opening hours	*p* = 0.77	*p* = 0.56	*p* = 0.65
Wait for appt.	*p* = 0.06	*p* = 0.25	*p* = 0.14
Availability	*p* = 0.70	*p* = 0.13	*p* = 0.12
Wait for physician	*p* = 0.63	*p* = 0.08	*p* = 0.06
Quality of equipment	*p* = 0.91	*p* = 0.75	*p* = 0.53
Hotel aspects	*p* = 0.63	*p* < 0.05	*p* = 0.05
